# Willingness to Seek Help for Depression in Young African American Adults: Protocol for a Mixed Methods Study

**DOI:** 10.2196/16267

**Published:** 2020-02-11

**Authors:** Benita A Bamgbade, Jamie C Barner, Kentya H Ford, Carolyn M Brown, William B Lawson, Kimberly Burdine

**Affiliations:** 1 Department of Pharmacy and Health Systems Sciences Bouve College of Health Sciences Northeastern University Boston, MA United States; 2 Division of Health Outcomes and Pharmacy Practice College of Pharmacy University of Texas at Austin Austin, TX United States; 3 Department of Psychiatry Dell Medical School The University of Texas at Austin Austin, TX United States; 4 Student Counseling Center The University of Texas at Dallas Dallas, TX United States

**Keywords:** African American young adults, depression, willingness to seek help, Theory of Planned Behavior

## Abstract

**Background:**

In the United States, among those living with mental illness, 81% of African American (AA) young adults do not seek treatment compared with 66% of their white counterparts. Although the literature has identified unique culturally related factors that impact help seeking among AAs, limited information exists regarding the development and evaluation of interventions that incorporate these unique factors.

**Objective:**

This study aims to describe a study protocol designed to develop a culturally relevant, theory-based, psychoeducational intervention for AA young adults; to determine if exposure to the intervention impacts AA young adults’ willingness to seek help; and to determine whether cultural factors and stigma add to the prediction of willingness to seek help.

**Methods:**

The Theory of Planned Behavior (TPB) and Barrera and Castro’s framework for cultural adaptation of interventions were used as guiding frameworks. In stage 1 (information gathering), a literature review and three focus groups were conducted to identify salient cultural beliefs. Using stage 1 results, the intervention was designed in stage 2 (preliminary adaptation design), and in stage 3 (preliminary adaptation tests), the intervention was tested using pretest, posttest, and 3-month follow-up surveys. An experimental, mixed methods, prospective one-group intervention design was employed, and the primary outcomes were participants’ willingness and intention to seek help for depression and actual help-seeking behavior.

**Results:**

This study was funded in May 2016 and approved by the University of Texas at Austin institutional review board. Data were collected from November 2016 to March 2016. Of the 103 students who signed up to participate in the study, 70 (67.9%) completed the pre- and posttest surveys. The findings are expected to be submitted for publication in 2020.

**Conclusions:**

The findings from this research are expected to improve clinical practice by providing empirical evidence as to whether a culturally relevant psychoeducational intervention is useful for improving help seeking among young AAs. It will also inform future research and intervention development involving the TPB and willingness to seek help by identifying the important factors related to willingness to seek help. Advancing this field of research may facilitate improvements in help-seeking behavior among AA young people and reduce the associated mental health disparities that apparently manifest early on.

**International Registered Report Identifier (IRRID):**

DERR1-10.2196/16267

## Introduction

### Background

As the leading cause of disability in the United States, depression impacts 15.7 million US adults and represents a significant health problem [1,2]. Although lifetime prevalence of depression is higher among whites compared with African Americans (AAs), 17.9% and 10.4%, respectively, AAs live with significantly more persistent, chronic, impairing, and disabling depression compared with whites [3,4].

Despite high levels of persistent and disabling depression, treatment among AAs is suboptimal. This higher disease burden is especially concerning when disparities in mental illness treatment are considered. There is evidence that AAs are less likely to receive treatment compared with whites [5,6]. It is estimated that 70% of AA adults living with mental illness received no treatment compared with 53% of white adults [5]. Moreover, lack of help seeking among young AA adults was higher, with 81% receiving no treatment, compared with 66% of young white adults [5]. Therefore, despite an estimated lower prevalence of depression, AAs live with more persistent, chronic, and disabling depression that is more likely to be untreated.

The literature identifies lack of perceived need, financial costs, the desire to handle the illness on one’s own, and stigma as common reasons why US adults do need to seek help [7-9]. Although these factors impact help seeking across racial and cultural lines, the literature has also identified unique cultural factors that impact help seeking among AA adults and young adults. These unique cultural factors include culturally embedded stigma, attitudes toward treatment, the influence of family and friends, medical mistrust, self-reliance, and religiosity [10-25]. To date, much of the literature evaluating these unique cultural factors have been descriptive in nature. Research is needed to address the influence of culturally-linked barriers and interventions targeting culturally linked barriers that may impact help seeking. If left unaddressed, the lack of help seeking among AAs may lead to greater disparities in depression treatment, which could lead to suboptimal outcomes such as suicide [26,27], which is the third leading cause of death among AA young adults (aged 18-25 years) [28].

Psychoeducational interventions have been identified as a tool to improve mental health help seeking. Psychoeducational interventions are a type of psychosocial treatment that combines psychotherapeutic and educational interventions using a collaborative and patient empowerment approach [29,30]. These interventions have been delivered in person, over the phone, through written patient information, and over the internet [31-34]. Psychoeducational interventions typically provide factual didactic information and can also include interactive activities and consumer educators. Psychoeducational interventions have demonstrated utility in preventing major depressive disorder [35], decreasing symptom burden [35-37], decreasing the risk of depression relapse [37], improving the quality of life [36], and improving global functioning [37]. Mental Health First Aid (MHFA) is such an intervention and is recognized as a Substance Abuse and Mental Health Services Administration (SAMHSA) national evidence-based program. It has demonstrated effectiveness in increasing mental illness knowledge, decreasing stigma, and improving help-seeking intentions and behaviors [33,38,39]. MHFA is an 8-hour course designed to provide members of the community with skills to assist during a mental health crisis and to help someone who may be developing a mental health problem [39]. MHFA is framed by two theoretical principles: (1) stigma can be reduced through awareness raising and education and (2) social support is instrumental in reducing risk for mental illness and in assisting a person with a mental health problem [40,41]. In addition to its standard form, MHFA has been successfully adapted for other populations (eg, rural populations and Aboriginal Australians) [33,42-46]; however, no such adaptation exists for AAs.

Despite the success of psychoeducational interventions and the identification of cultural factors that are unique to AAs, limited research has addressed the influence of culturally-linked barriers and interventions targeting culturally linked barriers that may impede help seeking [13,32,47]. Interventions in the literature report limited success and have typically not documented the utilization of a theoretical foundation for intervention design. Therefore, a gap currently exists regarding culturally relevant, theory-based psychoeducational interventions for AAs. Research is needed to address this gap, as this type of intervention represents an opportunity to target culturally embedded stigma and other culturally related factors and improve help seeking among a vulnerable and at-risk population. Furthermore, considering that AA young adults are more likely to not seek help and that suicide represents the third leading cause of death among AA young adults (aged 18-25 years) [28], this subgroup represents an even more vulnerable and at-risk population. In addition, most literature evaluating cultural factors related to help seeking and psychoeducational interventions in AAs have focused on AA adults, leaving cultural factors and interventions among AA young adults less understood. Given the factors related to AA help seeking (eg, attitudes, cultural factors, family and friends, and barriers), the Theory of Planned Behavior (TPB) is the proposed mechanism of action and guiding framework for this study.

### Objectives

The overall objective of this study is to understand how a culturally relevant, theory-based, interactive psychoeducational intervention can impact depression help-seeking willingness and subsequent behavior among AA college students. The central hypothesis is that the culturally adapted psychoeducational intervention will significantly improve willingness to seek help for depression.

Specifically, the aims are as follows:

- Aim 1: To develop a culturally relevant, theory-based, interactive psychoeducational intervention for AA college students. The intervention, guided by MHFA, a SAMHSA National Evidence-Based Program, was created through incorporating findings from the literature and qualitative focus groups.
- Aim 2: To determine if exposure to a culturally relevant psychoeducational intervention impacts AA students’ willingness to seek help, attitude toward seeking help, perceived behavioral control over seeking help, depression stigma, and actual help-seeking behavior. We hypothesized that after the intervention, participants would be more willing to seek help for depression, have more favorable attitudes, and report increased perceived behavioral control and less stigma regarding depression.
- Aim 3: To determine whether stigma and the cultural variables (ie, medical mistrust, self-reliance, and religiosity) add to the prediction of the TPB constructs (ie, attitude, subjective norm, and perceived behavioral control) in predicting AAs’ willingness to seek help.

## Methods

### Intervention

**Figure 1:**
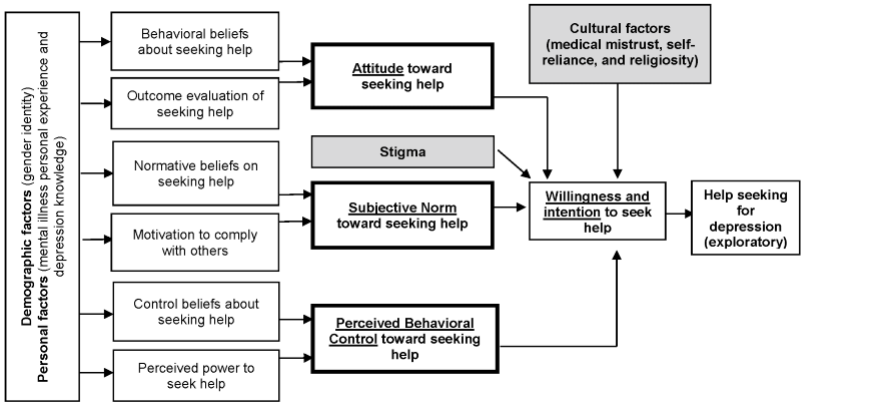
Conceptual model: using the Theory of Planned Behavior to predict willingness to seek help.

The study intervention was developed using the TPB and Barrera and Castro’s [48] framework for cultural adaptation of interventions. The TPB posits that behavioral intention directly predicts actual behavior (see Figure 1 for study conceptual model) [49]. The TPB is an expectancy value–based attitude-behavior model designed to explain behaviors in which people are able to exert self-control [49,50]. In this study, behavioral intention or willingness to seek help for depression is determined by attitudes toward seeking help, subjective norms associated with seeking help, and perceived behavioral control over seeking help. Attitude, subjective norm, and perceived behavioral control are determined by composites of related beliefs. Attitude comprises behavioral beliefs and outcome evaluations. Subjective norm comprises normative beliefs and the motivation to comply with others. Finally, perceived behavioral control comprises control beliefs and perceived power. The TPB was selected as a guiding framework because of its success in examining factors that impact other various health-related behaviors (eg, condom use, exercise behavior, dietary behavior, breastfeeding, and health screenings) [51-55]. In addition, factors that impact AA help seeking that were identified in the literature closely align with the TPB variables (eg, attitude toward treatment, influence of family and friends, and barriers such as lack of knowledge). Studies using the TPB in mental health help seeking have reported models that explain 42% to 93% of the variance in willingness or intention [56-59]. Among these studies, although samples did include some AAs, no sample consisted solely of AAs. At present, limited TPB studies in AA-only samples exist; however, no known studies exist in the area of mental health care help seeking [60-63].

Barrera and Castro’s [48] cultural adaptation framework was also used as a guiding framework. It suggests that researchers and clinicians undertake the following steps to adapt an intervention: (1) information gathering, (2) preliminary adaptation design, (3) preliminary adaptation tests, and (4) adaptation refinement [48]. Researchers begin with the *information gathering* stage, which involves reviewing the literature and conducting quantitative or qualitative studies to understand and identify ideas that have the most potential to close the existing disparity gap. With a thorough understanding of the unique issues facing a particular population, researchers then draft an intervention adaptation (ie, *preliminary adaptation design*). Next, in the *preliminary adaptation tests* stage, researchers conduct pilot studies and use a mixture of quantitative and qualitative measures to evaluate the success of the adaptation. In addition, in this stage, researchers should assess “sources of program non-fit, implementation difficulties and difficulties with program content or activities.” [48]. Finally, in the *adaptation refinement* stage, data collected from the previous step are used to revise the intervention. This project focused on the *information gathering, preliminary adaptation design*, and *preliminary adaptation tests* stages. The results from this study, which will be available in 2020, will assist with the revision of the intervention in the *adaptation refinement* stage. The cultural adaptation steps related to this study are detailed in the following sections.

### Study Overview, Sample, and Recruitment

An experimental, mixed methods, prospective, pretest, immediate posttest, and 3-month follow-up, one-group intervention research design was employed. This study was approved by the University of Texas at Austin institutional review board. The study design included three stages (ie, information gathering, preliminary adaptation design, and preliminary adaptation tests). In the information gathering stage, a literature review and qualitative focus groups were conducted. The findings from this stage were used to design the intervention in the preliminary adaptation design stage. Finally, in the preliminary adaptation tests stage, the intervention was evaluated using a self-report paper survey administered immediately before (pretest) and immediately after (posttest) the intervention. After 3 months, a Web-based self-report survey was administered to all participants (3-month follow-up). Aim 1 was achieved during the information gathering and preliminary adaptation design stages, whereas aims 2 and 3 were achieved during the preliminary adaptation tests stage.

#### Study Sample

The study sample for the focus groups and intervention evaluation consisted of AA undergraduate college students enrolled at a southwestern US university. Students who participated in the focus groups were also invited to participate in the intervention. AA undergraduate students were selected for this study because of evidence showing greater disparities in treatment among AA young adults. In addition, because depression onset typically occurs in the mid-20s [64], engaging students in undergraduate education may strategically prepare them for potential depression onset in themselves and onset among their peers. Participants were eligible for this study if they (1) were aged 18 to 25 years, (2) self-identified as black or AA, (3) were enrolled as an undergraduate (part time or full time) student during the 2016 to 2017 academic year, and (4) had never been diagnosed with and/or received treatment for a mental health condition. Individuals who have received a diagnosis and received treatment for a mental health condition were excluded from the study because of the intervention purpose and the unique needs of these individuals. The intervention was designed to teach people how to identify mental illness and how to get help. Furthermore, there is evidence that individuals who have had personal experiences in mental health care and have disengaged from treatment require specialized interventions [65]. We determined that a sample of 55 was needed to detect a difference in the prediction of willingness to counsel when adding the cultural variables (alpha=.05, beta=.80, and effect size=0.15), with seven predictors (ie, attitude, subjective norm, perceived behavioral control, stigma, cultural mistrust, self-reliance, and religiosity).

#### Study Recruitment

There is evidence documenting suboptimal recruitment and participation of AAs in research studies and increased loss to follow-up [66-68]. In an effort to mitigate potential problems, this study leveraged relationships with historically black social fraternities to enroll participants. Local campus chapters hosted specific study segments (ie, focus group sessions and the intervention) as events during their fraternity week at the university. Fraternity weeks are weeks designated to specific fraternities on campus, where fraternities host a variety of events geared toward fraternity initiatives. In fact, one of the fraternities, Omega Psi Phi, has a mental health initiative called *Brother, You're on My Mind: Changing the National Dialogue Regarding Mental Health Among African American Men*, which is featured as part of the National Institute on Minority Health and Health Disparities [69]. In addition to Omega Psi Phi, the local fraternity chapter of Alpha Phi Alpha was also instrumental in supporting this event. Fraternity weeks are heavily attended by the general body of AA college students. Organization leaders received study flyers for distribution to their members and other men and women who met the study criteria. Study flyers were distributed during fraternity week promotion initiatives. Study flyers contained a description of the study and provided the primary researcher’s contact information. Interested students were asked to sign up via direct contact with the primary researcher (via email) or electronically through Google forms.

### Aim 1: Information Gathering

A literature review was conducted, and several factors impacting AA help seeking were identified, including attitudes toward treatment, influence of family and friends, lack of knowledge, stigma, medical mistrust, self-reliance, and religiosity [10-25]. In addition, three focus groups were conducted to further identify factors that impact help seeking among AA young adults. Saturation was reached after three focus groups. Focus groups used the TPB as a guiding framework to facilitate elicitation of the salient behavioral, normative, and control beliefs associated with seeking help for depression. Questions used to elicit these beliefs were all adapted using a publication by Ajzen [49,70]. Three 60- to 90-min focus groups were conducted with 8 participants per group. As depression is often viewed as a *taboo* topic, a self-write activity was used at the beginning of each focus group to ease students into the discussion. Participants were asked to write or draw a response to the question *What is depression?* and then to share their definitions. Following this, participants were asked questions to assess their behavioral, normative, and control beliefs related to seeking help for depression. Each participant received a US $30 gift certificate for their participation.

Focus groups were audio recorded and subsequently transcribed by a third party. A thematic analysis was conducted in light of the TPB constructs to identify emerging themes related to seeking help for depression using Braun and Clarke’s approach for thematic analysis [71]. The primary researcher and a qualitative expert worked together to identify the most frequently mentioned beliefs from the focus group analysis. According to the TPB, these beliefs represent students’ modal beliefs, which represent the salient beliefs of the population of interest and, therefore, were used in the surveys. The TPB also suggests that an individual has five to nine beliefs that he or she holds to be salient. This guideline, along with analyses of belief frequencies, was used to determine salient beliefs for this study.

### Aim 1: Preliminary Adaptation Design

Knowledge gained in the information gathering stage was used to develop the psychoeducational intervention and a related survey instrument (ie, preliminary adaptation design). The study intervention was developed using MHFA as a guiding framework [40,41]. The first MHFA principle (ie, stigma can be reduced through awareness raising and education) was central to section 1 of the intervention (Textbox 1), which included activities and presentations to raise awareness and encouraged participants to evaluate how they approach a physical illness versus how they would approach a mental illness. In addition, section 1 included information on depression prevalence, signs, and symptoms as well as available treatments and common culturally related depression myths. The second MHFA theoretical principle (ie, social support is instrumental in reducing the risk of mental illness and assisting a person with a mental health problem) guided how intervention presenters approached intervention materials and topic discussions such that the information provided would be applicable to participants as well as a friend or family member.

**Psychoeducational intervention outline.**

**Section 1: Pharmacist**

- Introduction: Discuss house rules and purpose of the project
- Large group discussions: Participants discuss a time when they were sick. Moderator reveals that participants only referenced physical symptoms and illnesses, sought help from a doctor, and underwent treatment and recovered.
- Active learning exercise: Fact or fiction—the pharmacist moderator reads a statement (eg, antidepressants are addictive), and participants hold up signs that read either fact or fiction. When disagreement occurs, the pharmacist leads a group discussion.
- Depression overview: Medical definition of depression, mental illness in the United States and among African Americans (prevalence and treatment statistics), and causes of depression and available treatments (psychotherapy and medication)
- Active learning exercise: Jelly in a jar—3 participant volunteers are asked to get jelly out of the jar using an oversized spoon. As they struggle to complete the task, the moderator provides commentary (eg, “I don’t think you are trying hard enough”). After the task, the moderator tells participants that this exercise illustrates what it is like when you tell someone to “just get over your depression.”

**Section 2: Licensed Psychologist**

- Video and stigma and cultural factors discussion: A young African American celebrity athlete discusses his personal experience with mental illness and his journey to recovery
- Psychotherapy overview and question and answer session: An African American psychologist from the university counseling center leads a discussion on the purpose of psychotherapy and what one might expect from engaging mental health services

**Section 3: Consumer Educator**

- Presentation and question and answer session: An African American college student living with major a depressive disorder and schizophrenia shares his lived experience with mental illness. He was also a consumer educator representative of the National Alliance on Mental Illness.

Using the aforementioned principles, the intervention consisted of three sections (see Textbox 1). Section 1 was led by the primary researcher, who is an AA and a licensed pharmacist. This section consisted of the introduction, an opening activity, an active learning activity (fact or fiction) designed to highlight and correct common myths about depression, an overview of depression, and another active learning activity (peanut butter in the jar) designed to illustrate what it feels like when someone tells a person with depression to “just get over it.” Section 2, led by a licensed AA psychologist (from the university health center and the liaison to black and AA students in the counseling and mental health center), focused on stigma and the unique cultural variables that impact AA help seeking (identified in the information gathering stage). This section also featured a video clip of a young AA celebrity athlete discussing his personal experience with mental illness and his journey to recovery, followed by a group discussion of the video and cultural issues related to depression help seeking and a psychotherapy question and answer session. The last section, section 3, was led by a young AA college student consumer educator from the National Alliance on Mental Illness. He shared his lived experience with schizophrenia and depression, which was followed by an interactive question and answer session. The study intervention lasted 2 hours and 30 min, with multiple active learning activities to facilitate participant engagement and reduce participant fatigue. At the conclusion of this one-time intervention, participants were provided with a list of campus and local mental health resources. This list included campus services specifically for AA students. Furthermore, participants were encouraged to share what they learned in the intervention with their family and other students.

### Aims 2 and 3: Preliminary Adaptation Tests

**Figure 2:**
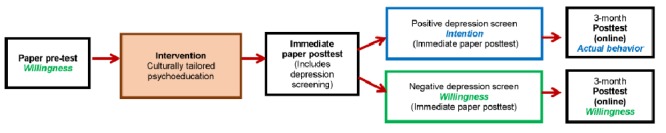
Data collection process.

According to Barrera and Castro’s [48] framework for cultural adaptation, the preliminary adaptation tests stage involves pilot studies to evaluate the success of the adaptation using both quantitative and qualitative measures. In this study, this stage was completed through implementing and assessing the intervention (Figure 2).

### Data Collection

Quantitative data, which were developed in accordance with the TPB and intervention content, were collected in the pretest, posttest, and 3-month follow-up surveys. Table 1 shows the variables that were collected for each survey. Posttest surveys included evaluation items that were quantitative using Likert-type scales and qualitative using open-ended responses. A pilot test of the questionnaire was conducted to assess the reliability and validity of the instrument with regard to all survey constructs. Feedback from the pilot testing was used to modify the final questionnaire, which was administered to the target population in the preliminary adaptation tests stage. Surveys were linked by a unique code created by each participant using an algorithm, thus rendering all surveys anonymous.

**Table 1:** Study variables by survey.

Pretest	Posttest	3-month follow-up
Willingness to seek help	Negative depression screening: willingness to seek help	Negative depression screening: willingness to seek help
	Positive depression screening: intention to seek help	Positive depression screening: help-seeking behavior
Attitude	Attitude	Attitude
Subjective norm	Subjective norm	Subjective norm
Perceived behavioral control	Perceived behavioral control	Perceived behavioral control
Depression stigma	Depression stigma	Depression stigma
Cultural variables: medical mistrust, self-reliance, and religiosity	Cultural variables: medical mistrust, self-reliance, and religiosity	Cultural variables: medical mistrust, self-reliance, and religiosity
Demographic and personal characteristics	Selected demographic and personal characteristics and intervention evaluation	Selected demographic and personal characteristics

#### Demographic and Personal Variables

Participants self-reported demographic (eg, gender identity and ethnicity) and personal (eg, personal and familial mental illness diagnosis, previous mental health course training, stress level, and extracurricular activity involvement) variables as well as depression knowledge. All variables were collected at pretest, and selected variables were collected at posttest and 3-month follow-up (eg, depression knowledge and stress level). Participants were also screened for depression at posttest using the 9-item Patient Health Questionnaire [72]. A cutoff score of greater than or equal to 10 was used for representing moderate depression to severe depression.

#### Theory of Planned Behavior Variables

Willingness, attitude, subjective norm, and perceived behavioral control were assessed at pretest, posttest, and 3-month follow-up. Willingness was measured at pretest for all participants because of the hypothetical nature of seeking help. Intention was only measured immediately after the intervention at post-test for participants who screened positively for depression. At pretest, although some participants did not have depression, others may not have known that they had depression; therefore, asking about their intention to seek help would have been inappropriate. Furthermore, once participants were screened, asking about their intention to seek help among those who screened negatively for depression would also have been inappropriate. Furthermore, participants who screened positively for depression at posttest were also asked to answer questions created by the authors regarding their help-seeking behavior at 3-month follow-up. All participants who screened negatively for depression were asked to answer questions regarding their willingness to seek help. Attitude, subjective norm, and perceived behavioral control were measured directly and indirectly according to the TPB. These measures were developed by the authors in accordance with the TPB. Direct measures represent a more global evaluation of the variable (eg, “If I screened positively for depression, most people important to me would think I should seek professional help”), whereas indirect measures are belief-based evaluations of the variable (eg, “If I screened positively for depression, my parents would think I should seek professional help”) [73]. Direct and indirect measures were assessed at pretest, posttest, and 3-month follow-up.

#### Stigma and Cultural Variables

Stigma and the cultural variables measured in this study include medical mistrust, self-reliance, and religiosity. Stigma was measured using the Depression Stigma Scale [74], and medical mistrust was measured using the suspicion subscale of the Group-Based Medical Mistrust Scale [75]. Self-reliance was measured using the self-reliance and affect regulation subscales of the Strong Black Woman Cultural Construct Scale [76]. Items were modified to be gender neutral. Finally, religiosity was measured using a scale developed for this study, comprising items derived from the literature [77-79].

#### Intervention Evaluation

Intervention evaluation items were a combination of quantitative and written qualitative measures, as recommended by Barrera and Castro [48]. Quantitative items included items such as “What is your overall rating of the first presenter?” and “What is your overall rating of the course?” Qualitative free-response items included “What was most impactful from the course?” and “How can we improve the course?”

### Data Analysis

#### Primary Outcomes

Willingness, intention, and help-seeking behavior represent the primary outcomes. Intention and behavior were only measured in participants who screened positively for depression at posttest. Intention was measured at posttest, whereas behavior was measured at 3-month follow-up. Willingness and intention scores will be averaged separately, with higher scores representing increased willingness or intention. Behavior will be categorized into professional help and nonprofessional help, then assessed with “yes, sought help” and “no, did not seek help.” The impact of the intervention with regard to willingness will be assessed using repeated measures analysis of variance. Descriptive statistics will be calculated for intention and behavior. Data will be analyzed using SAS (version 9.4).

#### Predicting Willingness to Seek Help

The predictor baseline TPB variables (ie, attitude, subjective norm, and perceived behavioral control), stigma, and the cultural variables will be entered into two hierarchical regression models, as described in our conceptual model (Figure 1). We selected hierarchical regression to allow for comparison of the amount of variance explained in willingness from the cultural variables and stigma while controlling for the TPB variables. Model 1 will pre-test predictor indirect TPB variables (ie, indirect attitude, indirect subjective norm, and indirect perceived behavioral control), cultural variables (ie, medical mistrust, self-reliance, and religiosity), and stigma. Model 2 will consist of these same variables, except it will use the baseline predictor direct TPB variables (ie, direct attitude, direct subjective norm, and direct perceived behavioral control). We hypothesize that attitude, subjective norm, perceived behavioral control, stigma, and the cultural variables would explain a significant proportion of variance in willingness to seek help and that stigma and the cultural variables would significantly add to the prediction of willingness to seek help for depression.

#### Intervention Evaluation

Descriptive statistics will be calculated for each quantitative intervention evaluation item. Qualitative intervention items will be analyzed via thematic analysis to identify emerging themes regarding the most impactful part of and ways to improve the intervention. The frequency of each code will also be documented.

## Results

This is a study protocol. Analysis and presentation of results will be available in 2020. This study was funded in May 2016 and approved by the Institutional Review Board of the University of Texas at Austin in November 2016. Data were collected from November 2016 to March 2016. Of the 103 students who signed up to participate in the study, 70 (67.9%) completed the pre- and posttest surveys. The primary outcome is participants’ willingness and intention to seek help for depression. The secondary outcomes include attitude toward seeking help, perceived behavioral control over seeking help, and depression stigma.

## Discussion

### Significance and Challenges of the Research

This study takes the important initial step in developing a targeted and evidence-based psychoeducational program for young AAs. The findings from this research are expected to improve clinical practice by providing empirical evidence as to whether a culturally relevant psychoeducational intervention is useful for improving help seeking among young AAs. It will also inform future research and intervention development involving the TPB and willingness to seek help by shedding light on the relationship between important factors related to willingness to seek help. Advancing this field of research will be a step closer to improving help-seeking behavior among AAs and reducing AAs’ mental health care disparities, which may result in decreased mortality and morbidity and improved quality of life among AAs with untreated mental illness.

Over the course of this study, a significant challenge was recruiting participants. With prior experience and considering evidence documenting suboptimal recruitment and participation and increased loss to follow-up of AAs in research studies, we employed a unique recruitment strategy [66,67]. During the project design, we decided to recruit participants through historically black sororities and fraternities. Initial contact with fraternity and sorority leadership via email was unsuccessful. The primary researcher then joined and engaged with the Black Presidents’ Council, an organization of black student organization leaders on campus. Through involvement in the council, the primary researcher learned about fraternity and sorority interests, events, and priorities and developed relationships with organization leaders. Through these relationships, the primary researcher began collaborating with fraternity organization leaders on how to meet organizational priorities and recruit for the study. Through these conversations, the recruitment strategy of using fraternity weeks to host specific study segments (ie, focus group sessions and the intervention) was developed. The relationships and collaborations formed significantly improved our recruitment strategy and retention.

### Limitations

This study is not without limitations. Our study sample consists of AA college students at a predominately white institution. Our results may not be generalizable to other AA young adults or AA adults. More research is needed to explore how this intervention may impact AA young adults who are not in college or who may attend a historically black college or university. Another limitation is the absence of a control group. A control group, in which an untailored intervention could have been used, would have strengthened the internal validity of this study. This was the initial study design; however, in the interest of achieving adequate power and in light of literature documenting difficulty in recruitment and retention of AAs in research studies, the authors elected to conduct a one-group intervention study. Therefore, our results may not truly reflect the impact of culturally adapting an intervention and may be similar to results from an unadapted psychoeducational intervention. It may be useful to use a control group when the intervention is further tested in Barrera and Castro’s [48] adaptation refinement stage. In addition, it is possible that students with a particular interest in mental health volunteered for the study, resulting in selection bias. Given the sensitive nature of mental illness and this study’s self-report surveys, response bias in the form of social desirability may have occurred. However, this may have been mitigated by the unique algorithm that participants created, rendering the surveys anonymous.

## Abbreviations

AA: African American
MHFA: Mental Health First Aid
SAMHSA: Substance Abuse and Mental Health Services Administration
TPB: Theory of Planned Behavior
